# Genomic characterization of multidrug-resistant clinical *Acinetobacter baumannii* isolates from a hospital in Paraguay

**DOI:** 10.3389/fcimb.2025.1620479

**Published:** 2025-07-31

**Authors:** Sandra Sánchez-Urtaza, Laura Alfonso-Alarcón, Rocío Arazo del Pino, Tessa Burgwinkel, Alain Ocampo-Sosa, Ruth Gonzalez, Kyriaki Xanthopoulou, Paul G. Higgins, Itziar Alkorta, Lucia Gallego

**Affiliations:** ^1^ Laboratory of Antibiotics and Molecular Bacteriology, Department of Immunology, Microbiology and Parasitology, Faculty of Medicine and Nursing, University of the Basque Country, Leioa, Spain; ^2^ Institute for Medical Microbiology, Immunology and Hygiene, Faculty of Medicine and University Hospital Cologne, University of Cologne, Cologne, Germany; ^3^ German Center for Infection Research (DZIF), Partner Site Bonn-Cologne, Cologne, Germany; ^4^ Microbiology Service, University Hospital Marqués de Valdecilla, Health Research Institute (Instituto de Investigación Valdecilla), Santander, Spain and CIBERINFEC, Instituto de Salud Carlos III, Madrid, Spain; ^5^ National Hospital of Itaugua, Itaugua, Paraguay; ^6^ Department of Biochemistry and Molecular Biology, Faculty of Science and Technology, University of the Basque Country, Leioa, Spain

**Keywords:** *Acinetobacter baumannii*, Paraguay, whole genome sequencing, epidemiology, antibiotic resistance, plasmids

## Abstract

*Acinetobacter baumannii* is a clinically important pathogen capable of causing serious nosocomial infections and acquiring resistance to antimicrobials, particularly carbapenems, making treatment difficult and prolonging hospital stays. In Latin America, high carbapenem-resistance rates have been described among *A. baumannii* isolates, however, Paraguay is one of the countries with limited data in this regard. Therefore, we aimed to investigate resistance rates of *A. baumannii* isolates from the National Hospital of Itaugua (NHI), Paraguay, from their database of 2022, and from December 2023 to February 2024, and to study in detail a representative group of multidrug-resistant clinical isolates. For this purpose, data were analyzed considering diagnostic, sample type and antimicrobial susceptibility. Eight *A. baumannii* isolates recovered from patients in six separate ICUs in 2024 were then selected and subjected to susceptibility testing using VITEK^®^ and to short- and long-read sequencing, and clonality, resistome, virulome and plasmidome of the isolates were investigated. IC2 (ST2 Pasteur, ST1816/195 Oxford and ST872 Oxford) was the predominant clone among the Paraguayan isolates, and a single isolate belonging to clone IC5 (ST79 Pasteur and ST1283 Oxford) was also identified. The carbapenemase gene *bla*_OXA-23_ was located in transposons Tn*2006* and Tn*2008*. Additionally, other antibiotic resistance genes conferring resistance to aminoglycosides, macrolides, sulfonamides, chloramphenicol, tetracyclines and trimethoprim were identified, and were found embedded in genetic environments containing mobile genetic elements. Multiple virulence genes were also detected, mainly promoting biofilm formation and immune system modulation. Plasmid analysis showed the presence of plasmids ranging in size from 2.27 to 10.74 Kb. This work describes the dissemination of the emerging clone IC2 in Paraguay and offers a detailed analysis of the resistome, virulome and plasmidome of carbapenem-resistant *A. baumannii* strains. The results obtained highlight the importance of correctly characterizing these multidrug-resistant pathogens to develop infection prevention and control strategies at hospital level.

## 1 Introduction

*Acinetobacter baumannii* is a highly clinically relevant pathogen that can lead to fatal nosocomial infections affecting mainly compromised patients or patients with underlying diseases. Infections caused by *A. baumannii* include pneumonia due to mechanical ventilation, catheter associated infections, bacteremia, urinary tract and bone infections, skin and wound infections. *A. baumannii* has a propensity to cause outbreaks at Intensive Care Units (ICU), and is strongly linked to the multidrug resistance phenotype, particularly carbapenem-resistance. Its clinical success is mainly due to its high virulence and the ability to survive in adverse conditions such as inanimate surfaces, combined with its desiccation and biocide tolerance, but also its capacity to acquire antibiotic resistance genes that are often coded by mobile genetic elements facilitating their spread (Jeon et al., 2023; Lee et al., 2017). Especially worrying is resistance to last resort antibiotics such as carbapenems, that usually makes infections difficult to treat, prolonging the hospital stay and making it necessary the use of more harmful and expensive antibiotics, leading sometimes to the death of the patients (Castanheira et al., 2023; Hamidian and Nigro, 2019; Kyriakidis et al., 2021; Ramirez et al., 2020; Vijayakumar et al., 2019).

During the COVID-19 pandemic, carbapenem resistant *A. baumannii* (CRAB) infections increased due to the massive admission of patients into hospitals, globalization, migration and the inappropriate use of antimicrobials (Castanheira et al., 2023; Montrucchio et al., 2022; Müller et al., 2023). In 2017 the World Health Organization published a list of critical pathogens for which new antibiotics were needed, with CRAB as the number one critical priority pathogen, and is still heading the list in the updated list published in 2024 (Tacconelli et al., 2018; World Health Organization, 2024). These traits contribute to its dissemination and contribute to its propensity for nosocomial outbreaks, especially where there are gaps in the infection-prevention and control regimes making it a critical pathogen threatening global health (Castanheira et al., 2023; Hamidian and Nigro, 2019; Montrucchio et al., 2022; Rodríguez et al., 2018).

According to Rodríguez et al. analyzing data obtained from 2000 to 2013, only 60% of the Latin-American countries had reported CRAB, mainly in Argentina and Brazil (Rodríguez et al., 2018). Resistance to carbapenems (meropenem and imipenem) in Latin-America is especially alarming considering it is ranging from 80 to 90%. The data obtained after the COVID-19 pandemic raised concern about the health system since that trend was increased in several countries such as Peru and Argentina, but also in Paraguay, Guatemala, Bolivia, El Salvador, Nicaragua and Ecuador (Manobanda Nata and Jaramillo Ruales, 2023). Most of the carbapenemase-encoding genes circulating in Latin-America belong to the oxacillinase families *bla*_OXA-23_, *bla*_OXA-58_, *bla*_OXA-24/40_ (*bla*_OXA-72_), *bla*_OXA-143_ (*bla*_OXA-253_) and *bla*_OXA-214_ (*bla*_OXA-215_, *bla*_OXA-264_, *bla*_OXA-265_, *bla*_OXA-575_) (Manobanda Nata and Jaramillo Ruales, 2023; Naas et al., 2017). However, less frequently, metallo-beta-lactamase genes such as *bla*_NDM_, *bla*_VIM_ and *bla*_IMP_ are also reported (Manobanda Nata and Jaramillo Ruales, 2023). Although Paraguay is one of the Latin-American countries with limited accessible data regarding antibiotic resistance and epidemiology of the circulating *A. baumannii* isolates, the most recent reports showed that 70% of the isolates are carbapenem-resistant (Melgarejo-Touchet et al., 2021) usually carrying *bla*_OXA-23-like_ and *bla*_NDM-like_ genes, while coexistence of *bla*_NDM-like_ and *bla*_OXA-58-like_ genes was reported in a 2% of isolates (Melgarejo-Touchet et al., 2021). International clone (IC) 5 and IC7 are the most common lineages found in the country (Rodríguez et al., 2016).

The aim of this work was the analysis of the resistance to antibiotics during the years 2022, 2023 and 2024, and the molecular characterization by whole genome sequencing of a representative series of carbapenem-resistant *A. baumannii* isolates recovered from the National Hospital of Itaugua (NHI), Paraguay, in 2024.

## 2 Materials and methods

### 2.1 Hospital database analysis and selection of bacterial isolates

The NHI is a specialized hospital affiliated to the Ministry of Public Health and Social Wellness, with more than 750 beds, from which 115 belong to the Intensive Care Units (ICUs). It is a reference center giving service to the Paraguayan population with the aim of providing preventive and curative health care. The data from 41 ICU isolates identified as *A. baumannii* complex and recovered between December 2023 and February 2024 were analyzed. For this study, within this last group, the first isolate recovered from each ICU (eight isolates in total) were selected for further analysis (**Table 1**). Furthermore, the evolution of antimicrobial resistance rates was analyzed based on the last official report of the NHI from 2022, which included information about diagnostics, type of clinical sample, species and antimicrobial susceptibility testing.

**Table 1:** Overview of the selected *A. baumannii* complex isolates recovered from National Hospital of Itaugua of Paraguay.

Isolate	Patient’s age	Patient’s sex	Sample type	Ward	Species identification (VITEK 2)	Isolation date	Genbank accession number
PR1	56	F	Tracheal secretion	RICU1	*A. baumannii* complex	06/02/2024	CP179709-CP179710
PR2	72	M	Tracheal secretion	AICU	*A. baumannii* complex	03/01/2024	CP179707-CP179708
PR3	6	F	Tracheal secretion	PICU	*A. baumannii* complex	14/01/2024	CP179705-CP179706
PR4	77	M	Tracheal secretion	RICU3	*A. baumannii* complex	05/02/2024	CP179702-CP179704
PR5	68	M	Tracheal secretion	RICU5	*A. baumannii* complex	07/01/2024	CP179699-CP179701
PR6	68	M	Tracheal secretion	RICU1	*A. baumannii* complex	07/02/2024	CP179696-CP179698
PR7	56	M	Urine	RICU4	*A. baumannii complex*	07/01/2024	CP179694-CP179695
PR8	87	M	Tracheal secretion	RICU4	*A. baumannii complex*	02/02/2024	CP179692-CP179693

F, Female; M, Male; PICU, Paediatric Intensive Care Unit; AICU, Adult Intensive Care Unit; RICU, Respiratory Intensive Care Unit.

### 2.2 Species identification and susceptibility testing

Species identification and antibiotic susceptibility testing were initially performed at the hospital using the automated method VITEK 2^®^ Compact (BioMérieux, Marcy l´Étoile, Francia) to determine the Minimum Inhibitory Concentrations (MIC) following the guidance of the Clinical and Laboratory Standards Institute (CLSI) (Lewis and Clinical and Laboratory Standards Institute, 2024). The species were later confirmed by *gyrB* multiplex PCR and PCR of the intrinsic *bla*_OXA-51_ gene, as previously described (Higgins et al., 2010b; Turton et al., 2006) and by whole genome sequencing. Isolates were determined to be MDR (Multidrug-Resistant) if they were at least resistant to one agent in three or more antimicrobial categories as previously described (Magiorakos et al., 2012).

### 2.3 Preliminary screening of carbapenemase genes

To investigate the presence of carbapenem-resistance genes, multiplex PCR experiments targeting *bla*_OXA-51_ *, bla*_OXA-23_ *, bla*_OXA-24/40_ *, bla*_OXA-58_ *, bla*_OXA-143_ and *bla*_OXA-235_ genes were first performed as previously described (Woodford et al., 2006; Higgins et al., 2010a, 2013). Additionally, two multiplex PCR were also carried out to detect the presence of other carbapenemases such as *bla*_VIM_ *, bla*_KPC_ *, bla*_NDM_ *, bla*_OXA-48_ *, bla*_IMI_ *, bla*_GES_ *, bla*_GIM_ and *bla*_IMP_ (Cerezales et al., 2021). The presence of these genes was further confirmed by whole genome sequencing.

### 2.4 Whole genome sequencing

For short-read sequencing, total DNA was purified with the DNeasy UltraClean Microbial Kit (Qiagen, Hilden, Germany) and libraries were prepared using the NEBNext^®^ Ultra™ II FS DNA Library Prep Kit (New England Biolabs, Ipswich, Massachusetts, USA) for a 250 bp paired-end sequencing on an Illumina^®^ MiSeq (Illumina, Inc., San Diego, California, USA). For long-read sequencing, genomic DNA was extracted using the NEB^®^ Monarch genomic DNA purification kit and DNA concentrations were measured with the Qubit fluorimeter (Thermo Fisher Scientific) using the Qubit™ 1X dsDNA Broad-Range Assay-Kit (Waltham, Massachusetts, USA). Libraries were prepared with the Rapid Barcoding Kit 24 V14 (SQK-RBK114.24) and sequenced on a MinION Mk1b device using a R10.4.1 (FLO-MIN114) flow-cell (Oxford Nanopore Technologies, Cambridge, UK). Hybrid genome assemblies using short- and long-reads were performed with Unicycler v0.5.1 (Wick et al., 2017) and genome annotation was assessed with Bakta web v1.9.1; DB:5.0.0 (Schwengers et al., 2021) and manually curated. The closed genome assemblies of the isolates generated for this study were deposited and can be found in GenBank under the BioProject ID PRJNA1197744 and the accession numbers of each isolate are detailed in **Table 1**.

#### 2.4.1 Analysis of the molecular epidemiology

Clonal relatedness was investigated by Multi Locus Sequence Typing (MLST) following Pasteur and Oxford schemes using the PubMLST website (Jolley et al., 2018), and analyzing the core genome MLST (cgMLST) using the Ridom SeqSphere+ software version 10.0.5 (Ridom GmbH, Münster, Germany) based on a core genome of 2390 alleles (Jünemann et al., 2013). A minimum spanning tree was also generated using Ridom SeqSphere+ to visualize the results.

#### 2.4.2 Investigation of antibiotic resistance and virulence genes

Antimicrobial resistance genes were detected using ResFinder v4.5.0 (Zankari et al., 2012) and the BetaLactamase DataBase (BLDB) (Naas et al., 2017), virulence factors were screened using Virulence Factor Database (VFDB) search tool (Liu et al., 2018) and Kaptive v2.0.1 (Cahill et al., 2022) while BLAST search against NCBI database (Camacho et al., 2009) was used for confirmation. Assembled genomes were analyzed in detail, edited and visualized using SnapGene Viewer 7.2.1 (www.snapgene.com) to look for insertion sequences and transposons associated with the antibiotic resistance genes using ISFinder version 2024-06-13 (http://www-is.biotoul.fr) and TnCentral: a Prokaryotic Transposable Element Database (Ross et al., 2021), respectively.

#### 2.4.3 Plasmid analysis

To investigate the plasmid content, plasmid extractions were first performed using the GeneJET Plasmid Miniprep Kit (ThermoFisher Scientific, Waltham, Massachusetts, USA) following the manufacturer’s instructions, and then loaded onto a 0.7% agarose gel. For further analysis, determination on the presence and classification of replicase genes was assessed by performing *A. baumannii* PCR-Based Replicon Typing, as previously described (Bertini et al., 2010), and doing a BLAST of the genomes against the Plasmid Typing Database developed by Lam et al (Lam et al., 2023).

### 2.5 Phenotypic analysis of virulence factors

#### 2.5.1 Biofilm formation experiments

Biofilm production was tested in biological triplicates, on three independent days following the protocol described by O' Toole and Kolter with slight modifications (O’Toole and Kolter, 1998). Briefly, overnight cultures were adjusted to 0.5 McFarland, and 100 μL of each culture were placed on a 24-well flat-bottom plate (Sarstedt^®^, Nümbrecht, Germany) with 900 μL of Mueller Hinton broth, and incubated overnight at 37°C. Culture media were then discarded, washed twice with 1 mL of distilled water and air-dried for 20 min. The staining was done by adding 1 mL of 0.7% crystal violet solution and incubating for 12 min at room temperature, and then, it was washed and air-dried as previously described. Stained biofilms were solubilized using 1 mL of 33% acetic acid solution and incubated with agitation for 5 min. Finally, 100 μL of each well were placed into a 96 well flat bottom plates to measure the absorbances at 600 nm on a Tecan Infinite M200 Pro Microplate Reader (Tecan Group Ltd., Männedorf, Switzerland). The absorbance value for crystal violet bound to uninoculated Müller Hinton Broth control wells was subtracted in order to correct the results for background staining. The results were expressed as the mean ± standard deviation of the replicates. *Escherichia coli* J53 and *Pseudomonas aeruginosa* PAO1 were used as negative and positive controls, respectively. The statistical analysis was performed using GraphPad Prism v8.0.2. Differences between the isolates and the control strains were analyzed using one-way ANOVA, followed by a Tukey *post hoc* test for multiple comparisons and statistically significant differences were defined as those with *p* < 0.05.

#### 2.5.2 Surface-motility assays

To investigate the motility of the isolates, overnight cultures were adjusted to 0.5 McFarland and 1 μL was placed in the center of the Motility Test Medium (Condalab, Madrid, Spain) plates. After 5 days of incubation at 37°C, plates were observed and compared to the positive and negative controls, *A. baumannii* ATCC 19606 and *A. baumannii* ATCC 17978, respectively.

## 3 Results

### 3.1 Hospital database analysis, characteristics and susceptibility testing of the isolates recovered from the NHI

In 2022, 11% of the microorganisms isolated in the hospital were *A. baumannii*, precisely, 84 isolates were recovered per month making a total of 1002 isolates, of which, 40% were isolated from female patients. Half of the isolates were recovered from respiratory samples and 18% from blood cultures (**Supplementary Figure S1A**). Analyzing the antimicrobial susceptibility rates of the year 2022, there was low susceptibility to imipenem and meropenem (15%), ceftazidime, ampicillin/sulbactam and ciprofloxacin (16%), gentamicin (37%) and amikacin (40%) (**Supplementary Figure S1B**). It is worth mentioning that those isolates resistant to carbapenems were also resistant to fluoroquinolones. In contrast, from December 2023 to February 2024, 88% of the *A. baumannii* isolates were recovered from respiratory samples and 3% from blood cultures (**Supplementary Figure S2A**). Antibiotic susceptibility analysis showed that 10% of isolates were susceptible to imipenem, meropenem, ceftazidime, ciprofloxacin and piperacillin/tazobactam; 24% were susceptible to amikacin and 100% were susceptible to colistin (**Supplementary Figure S2B**).

### 3.2 Species identification and preliminary screening of carbapenemase genes

The eight isolates selected for further analysis were identified as *A. baumannii* by amplification of the *gyrB* and *bla*_OXA-51_ genes. Investigation of carbapenemase genes showed that all the isolates were positive for *bla*_OXA-23._ No other carbapenemase-encoding genes were detected.

### 3.3 Analysis of the molecular epidemiology

**Figure 1:**
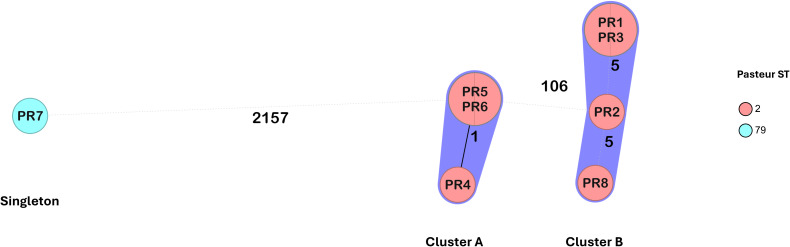
Minimum spanning tree of the *A. baumannii* isolates from the National Hospital of Itaugua (Paraguay) based on a core genome of 2390 alleles generated using Ridom SeqSphere+. The study IDs of the isolates are shown within the nodes. Isolates are colored by the assigned Pasteur STs.

Seven isolates belonged to International Clone 2 (IC2) and were assigned to ST2 according to the Pasteur scheme. Based on the Oxford scheme, isolates PR1, PR2, PR3 and PR8 were assigned to ST1816/ST195, while PR4, PR5, and PR6 were assigned to ST872. Notably, the assignment of two Oxford STs to some isolates was due to a duplication of the *gdhB* gene. Based on the cgMLST analysis, these seven isolates were grouped into two clusters (cluster A and cluster B), separated by 106 alleles (corresponding to ST872 and ST1816/ST195, respectively) (**Figure 1**). Within each cluster, isolates differed by five or fewer alleles. In cluster A, PR5 and PR6 differed from PR4 at a single locus, *ACICU_RS04400*, encoding an acetylglutamate kinase. In cluster B, isolates PR1 and PR3 differed from PR2 and PR8 at four loci: *ACICU_RS01055* (lytic transglycosylase domain-containing protein), *ACICU_RS07275* (hybrid sensor histidine kinase/response regulator), *ACICU_RS08455* (RND transporter), and *ACICU_RS11490* (alpha/beta hydrolase). Additionally, PR2 differed from PR1, PR3, and PR8 at *ACICU_RS13890* (hypothetical protein), while PR8 differed from the other cluster B isolates at four loci: *ACICU_RS02340* (lysine transporter LysE), *ACICU_RS03900* (hypothetical protein), *ACICU_RS13905* (aspartate aminotransferase), and *ACICU_RS14920* (metal-binding protein) (**Supplementary Table S1**). A singleton isolate, PR7, belonged to IC5 and was assigned to ST79 (Pasteur) and ST1283 (Oxford).

### 3.4 Investigation of antibiotic resistance and virulence genes

By whole genome sequencing the β-lactamase variants were confirmed (**Table 2**). Two variants of the *bla*_OXA-51-like_ gene were identified: *bla*_OXA-66_ (IC2) in seven isolates and *bla*_OXA-65_ (IC5) in isolate PR7. Two copies of the *bla*_OXA-23_ gene were also found in isolates PR1-PR6 and PR8. Regarding the chromosomal cephalosporinase genes, *bla*_ADC-73_ was identified in all the IC2 isolates, while *bla*_ADC-5_ was identified in isolate PR7, and the broad spectrum β-lactamase gene *bla*_TEM-1_ was also detected in the latter isolate.

**Table 2:** Overview of clonal lineages, sequence types and resistome of the *A. baumannii* isolates from the National Hospital of Itaugua.

Isolates	IC	Pasteur ST	Oxford ST	β-lactamase genes	Other antibiotic resistance genes
PR1	IC2	ST2	ST1816, ST195	*bla*_OXA-66_ *bla*_OXA-23_ *bla*_ADC-73_	*armA, aph(6)-Id, aph(3’’)-Ib, msr(E), mph(E), tet(B)*
PR2					*armA, aph(6)-Id, aph(3’’)-Ib, msr(E), mph(E), sul1, catA1, tet(B)*
PR3					
PR4			ST872		
PR5					
PR6					
PR7	IC5	ST79	ST1283	*bla*_OXA-65_	*aph(6)-Id, aph(3’’)-Ib, aadA1, aph(3’)-VIa, sat2, sul2, dfrA1*
				*bla*_OXA-23_	
				*bla*_TEM-1A_	
				*bla*_ADC-5_	
PR8	IC2	ST2	ST1816, ST195	*bla*_OXA-66_ *bla*_OXA-23_ *bla*_ADC-73_	*armA, aph(6)-Id, aph(3’’)-Ib, msr(E), mph(E), sul1, catA1, tet(B)*

IC, International Clone.

Other antibiotic resistance genes conferring resistance to aminoglycosides (*armA*, *aph(6)-Id*, *aph(3’’)-Ib, aph(3’)-Via, aadA1* and *sat2*), macrolides (*msr(E)* and *mph(E)*), sulfonamides (*sul1* and *sul2*), chloramphenicol (*catA1*), tetracyclines (*tet(B)*) and trimethoprim (*dfrA1*) were also identified (**Table 2**).

Regarding the virulome, all the isolates harbored a wide variety of genes involved in biofilm production, immune system modulation, secretion systems, acinetobactins and exotoxins (**Supplementary Figure S3**). Furthermore, capsule typing of the isolates revealed two different OC locus OCL1 in those isolates belonging to IC2, and OCL10 in the isolate belonging to IC5. Regarding the K locus, isolates within the IC2 cluster were subdivided into KL3 and KL81, and the IC5 isolate was categorized as KL9 (**Supplementary Figure S3**).

#### 3.4.1 Genetic environments of the β-lactamase genes

**Figure 2:**
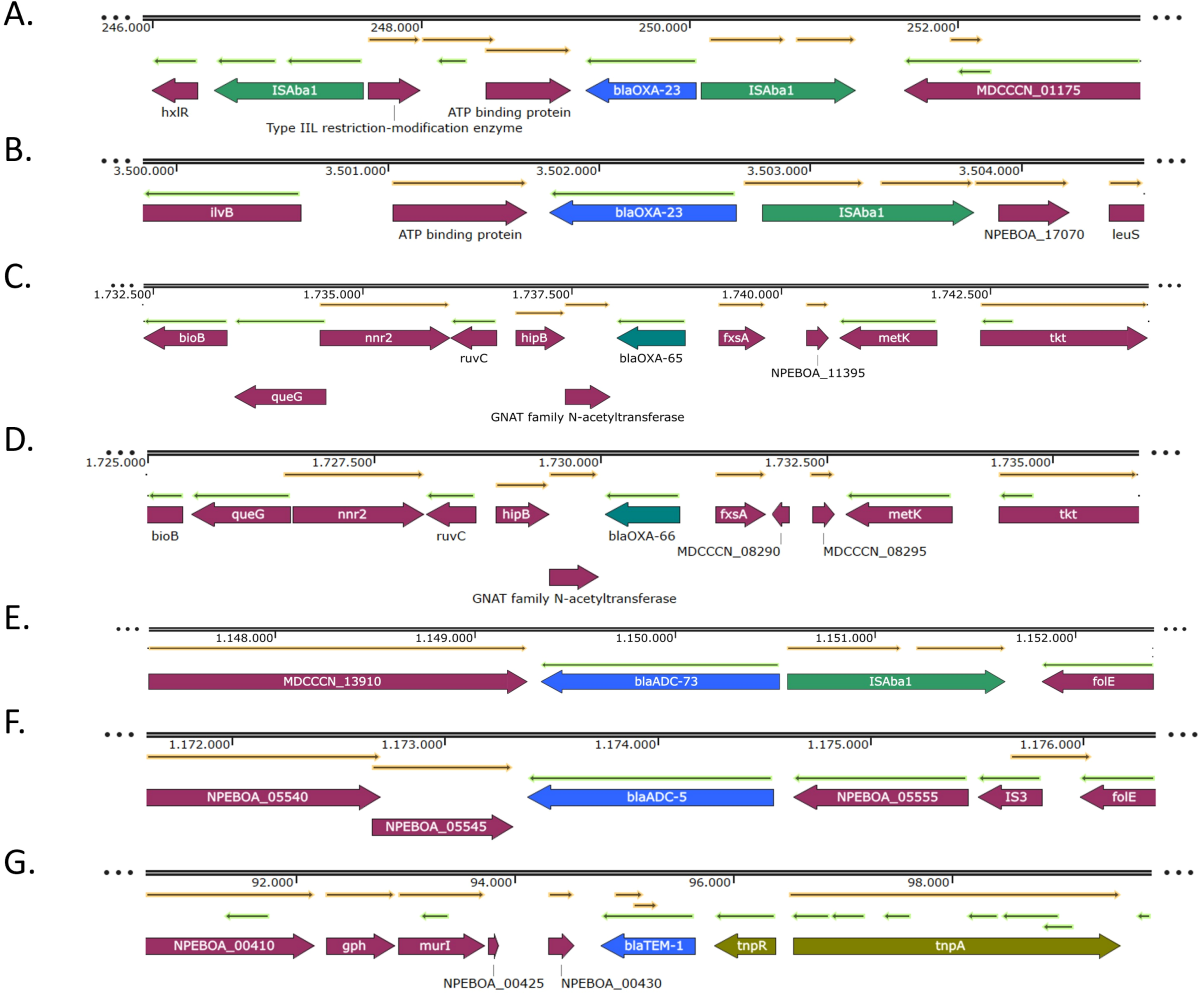
Genetic environments of the β-lactamase genes *bla*_OXA-23_ **(A, B)**, *bla*_OXA-51-like_ **(C, D)**, *bla*_ADC-like_ **(E, F)** and *bla*_TEM-1_ **(G)** genes. Arrows represent open reading frames, with colors indicating gene function: resistance genes in blue, mobile genetic elements in green, and other genes in purple. Arrow direction indicates transcriptional orientation.

The analysis and annotation of the genetic environment of the β-lactamase genes revealed that *bla*_OXA-23_ gene was located within different structures. In those isolates belonging to IC2 (PR1–PR6 and PR8), the gene was located within the transposon Tn*2006*, present in two identical copies per genome (**Figure 2A**). In the isolate belonging to IC5 it was harbored by the transposon Tn*2008* (**Figure 2B**). These transposons were structurally conserved and matched the canonical configurations in all the isolates. No insertion sequences were detected immediately upstream of the *bla*_OXA-51_ genes (**Figure 2C, D**). Regarding the intrinsic cephalosporinase genes, IS*Aba1* was upstream of *bla*_ADC-73_ gene (**Figure 2E**), while no mobile genetic element was found upstream of *bla*_ADC-5_ (**Figure 2F**). The *bla*_TEM-1_ gene was encoded in transposon Tn*3* (**Figure 2G**).

#### 3.4.2 Genetic environments of non-β-lactam antibiotic resistance genes

**Figure 3:**
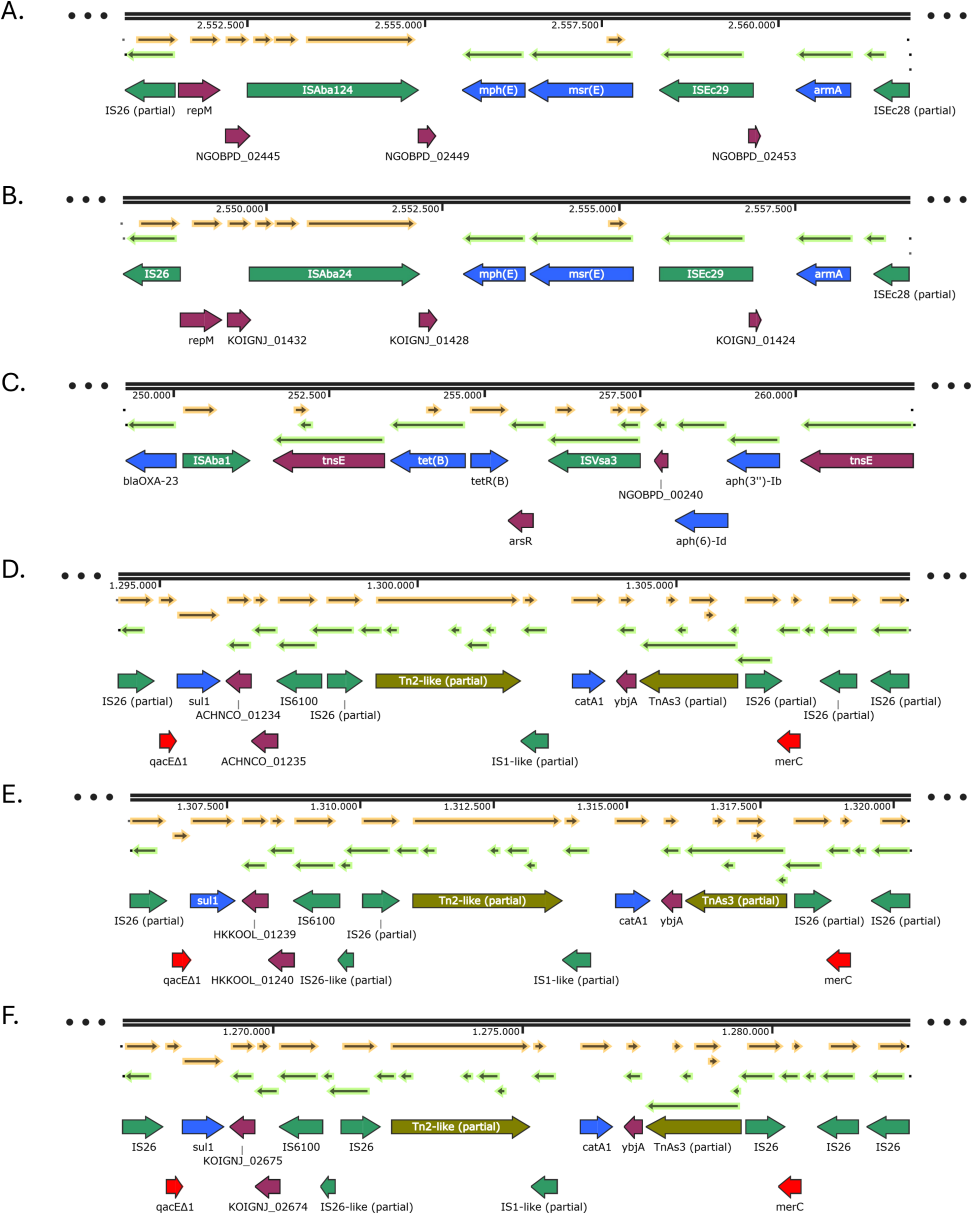
Genetic environments of other antibiotic resistance genes found in the IC2 isolates: *armA, msr(E)* and *mph(E)* **(A, B)**; *aph(3’’)-Ib, aph(6)-Id* and *tet(B)* **(C)**; *sul1* and *catA1* **(D-F)**. Arrows represent open reading frames, with colors indicating gene function: resistance genes in blue, mobile genetic elements in green, and other genes in purple. Arrow direction indicates transcriptional orientation.

The rest of the genes involved in resistance to other classes of antibiotics were also associated with mobile genetic elements. The *armA, mph(E)* and *msr(E)* genes were located on a Tn*6180*-derived fragment of the resistance island AbGRI3, flanked by partial sequences of IS*Ec28* and IS*26*. IS*Ec28* was positioned upstream of *armA*, followed by a permease gene, an IS*Ec29* inserted upstream of the macrolide resistance genes *mph(E)* and *msr(E)*, and an IS*Aba24* located downstream of those genes (**Figure 3A**). This structure was identical in isolates PR1 (three copies), PR2, PR3 (two copies), PR4 and PR8. In isolates PR5 and PR6, a single copy of the same structure was present, except that the IS*26* element was complete (**Figure 3B**). The aminoglycoside resistance genes *aph(3’’)-Ib* and *aph(6)-Id*, together with the tetracycline resistance gene *tet(B)*, were found in close proximity to the transposon Tn*2006* in all isolates belonging to IC2, presenting an AbGRI1-like resistance island structure. The insertion sequence IS*Vsa3* was identified downstream of *aph(3’’)-Ib* and *aph(6)-Id*, and upstream of *tet(B)*. Additionally, two copies of the *tnsE* gene, typically associated with Tn*7*-like transposons, were also detected downstream the *tet(B)* gene and upstream of the aminoglycoside resistance genes (**Figure 3C**).

A complex genetic environment harboring the resistance genes *sul1* and *catA1* was identified in the IC2 isolates (**Figures 3D-F**). In isolates PR2 and PR8, a ~15 kb resistance island was observed, including the genes *sul1, catA1, qacEΔ1* and *merC*, embedded within a mosaic of mobile genetic elements. The *sul1* gene was located downstream of the antiseptic resistance gene *qacEΔ1*, which in turn was preceded by a partial IS*26* element. Downstream of *sul1*, an IS*6100* element and another partial IS*26* element were present, followed by a partial Tn*2-like* transposon and a partial IS*1-like* insertion sequence. Further downstream and in the opposite orientation, the *catA1* gene was found, followed by *ybjA*, a partial Tn*As3* transposase gene, a partial IS*26* element, the mercury resistance gene *merC*, and two additional IS*26* partial sequences (**Figure 3D**). In isolates PR3 and PR4, a similar arrangement was identified, with a key difference, the IS*6100* element was interrupted by a partial IS*26-like* sequence, and one of the downstream IS*26* elements upstream of *merC* was absent (**Figure 3E**). In isolates PR5 and PR6, the gene arrangement was a combination of the two previously described contexts. In these isolates, the IS*26* sequences were complete, and IS*6100* was again interrupted by a partial IS*26-like* element. Additionally, two IS*26* copies were found upstream of the *merC* gene (**Figure 3F**).

**Figure 4:**
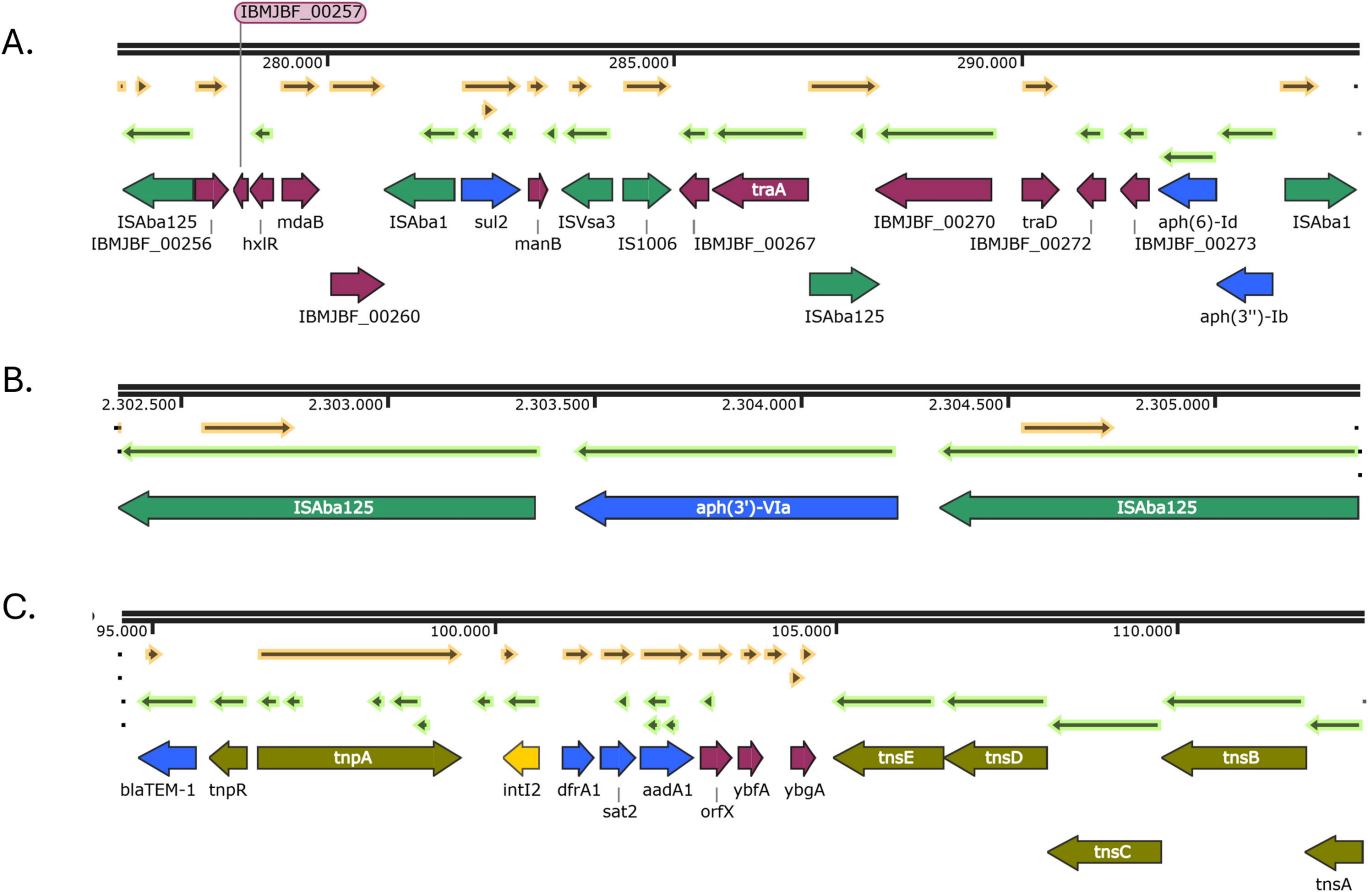
Genetic environments of other antibiotic resistance genes found in the IC5 isolate: *aph(6)-Id, aph(3’’)-Ib* and *sul2* **(A)**; *aph(3’)-VIa* **(B)**; *dfrA1, sat2* and *aadA1* **(C)**. Arrows represent open reading frames, with colors indicating gene function: resistance genes in blue, insertion sequences and transposons in green, integrons in yellow and other genes in purple. Arrow direction indicates transcriptional orientation.

In isolate PR7, the *aph(6)-Id* and *aph(3’’)-Ib* genes were located within the transposon Tn*5393c*, which includes an *ISAba1* element upstream of the aminoglycoside resistance genes. This transposon was embedded within a Tn*6250-like* structure that also carries the *sul2* gene. Notably, this Tn*6250* variant harbors an additional *ISAba125* element inserted between the *traA* and *traD* genes, which is not present in the canonical Tn*6250* structure (**Figure 4A**). Regarding the aminoglycoside resistance gene *aph(3’’)-VIa*, it was only present in isolate PR7, and was bracketed by two IS*Aba125* forming a transposon named Tn*aphA6* (**Figure 4B**). In addition, *dfrA1, sat2 and aadA1* genes were found in a typical class 2 integron structure, located between the Tn*7* transposition module (*tnsABCDE*) and the integrase gene *intI2*. Simultaneously, the Tn*3* transposon harboring the *bla*_TEM-1_ gene was embedded in the Tn*7* transposon forming a composite Tn*7*::Tn*3* structure (**Figure 4C**).

### 3.5 Plasmid analysis and location of the resistance genes

**Figure 5:**
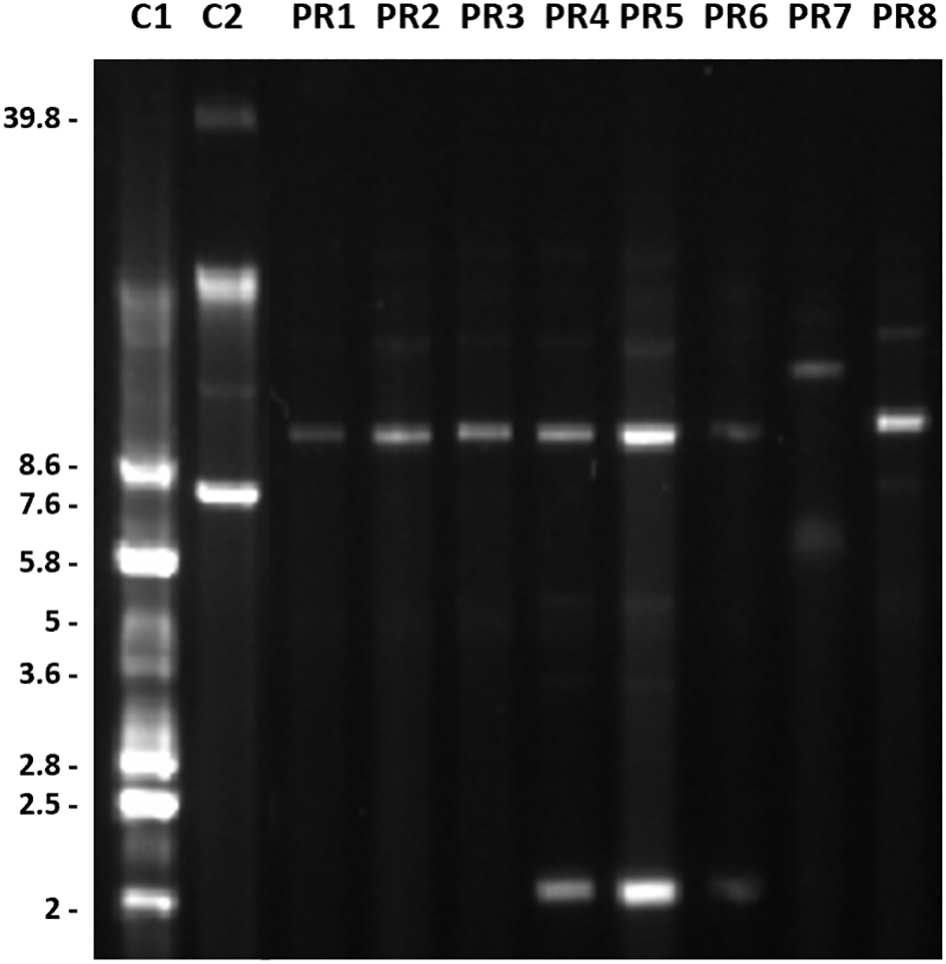
Plasmid profiles of the *A. baumannii* isolates recovered from the National Hospital of Itaugua, Paraguay. Molecular weights are expressed in Kb. Isolates C1, *E. coli* CECT 678 (NCTC 50193) and C2, *E. coli* CECT 679 (NCTC 50191) were used as molecular weight markers.

Conventional lysis experiments showed plasmid profiles including plasmids ranging in size from 2 to around 13.6 Kb (**Figure 5**). Sequencing further confirmed the presence of a plasmid of 8.73 Kb in all the isolates except PR7, an additional plasmid of 2.28 Kb in isolates PR4, PR5 and PR6; and a plasmid of 10.85 Kb in isolate PR7 were also detected.

PCR-based replicon typing experiments revealed the presence of replicases of group GR2 (Aci1/Aci2) in all the isolates except PR7, which was positive for a group GR4 (Aci4) replicase. Additionally, PR1-PR7 isolates were also positive for a replicase of group GR19 (rep135040). Replicon typing using the *Acinetobacter Plasmid Typing database* allowed us to type the 8.73 Kb plasmids as r3-T1 (Aci1) and the 2.27 Kb plasmids as r3-T15. The 10.84 Kb plasmid of isolate PR7 was typed as r3-T14. Additionally, we also detected a r3-T60 replicase gene in the contigs belonging to the chromosomes of the eight isolates, and that region had a higher read coverage than the rest of the chromosome. In order to confirm the integration of that plasmid in the chromosome and to exclude the possibility of an assembly error, long-reads were aligned to the hybrid assemblies to find a read long enough to analyze the genes flanking the replicase gene. The analysis of those genes showed that they were involved in metabolic processes that are usually coded by the chromosome in the reference strains ATCC 19606 and ATCC 17978, which allowed us to conclude the assembly was correct. Although the isolates were carrying at least one plasmid, no resistant genes were located within those structures, but toxin-antitoxin systems (*brnT/brnA*), septicolysin and TonB-dependent receptor *znuD2* genes were found.

### 3.6 Phenotypic analysis of virulence factors: biofilm production and motility

Statistically significant differences in biofilm production were observed among the isolates (One-way ANOVA, *p* < 0.0001), as shown in **Supplementary Figure S4**. All isolates except PR6 (*p* = 0.9932, Tukey’s *post hoc* test) exhibited significantly higher biofilm production compared to the negative control (*p* < 0.05). Notably, isolates PR1, PR4, and PR7 showed significantly greater biofilm production than the positive control (*p* < 0.05), indicating an enhanced biofilm-forming capacity.

All the isolates showed the same motility phenotype, more precisely, morphotype D according to the classification method described by Cosgaya et al (Cosgaya et al., 2019).

## 4 Discussion

Paraguay, like other Latin-American countries, has published little information on the incidence and epidemiology of *A. baumannii*. During 2022, an average of 743 microorganisms (bacteria and fungi) were recovered in the NHI, including isolates recovered upon hospital admission or and during hospitalization, of which 11% were identified as *A. baumannii*. Considering only the isolates recovered during hospitalization, *A. baumannii* is the most commonly isolated bacteria in the hospital. Thus, *A. baumannii* is of great concern to the healthcare community and it poses a challenge in the treatment of infections due to its high level of resistance to antibiotics (Rodríguez et al., 2018; Manobanda Nata and Jaramillo Ruales, 2023).

Carbapenem resistance rates of the *A. baumannii* isolates recovered in NHI in 2022 were above 80%. However, data obtained from the first two months of 2024 suggests that the carbapenem resistance rates are increasing up to 90% which is in accordance with data previously reported in the region (Melgarejo-Touchet et al., 2021; Manobanda Nata and Jaramillo Ruales, 2023). In the present study the majority of the *A. baumannii* isolates from the ICU patients were recovered from respiratory samples, which has also been reported in other studies, where isolates were recovered mainly from ICU patients that were suffering from pneumonia (Montrucchio et al., 2022; Brizuela Centurión et al., 2023; Castanheira et al., 2023).

Clonal relatedness analysis by MLST and cgMLST allowed us to identify the international clonal lineage to which the investigated isolates belong. A single isolate (PR7) was assigned to IC5 (ST79 Pasteur scheme), the so-called Pan-American clone that is characteristic and commonly described in CRAB isolates in Latin America, often along with clone IC7 (ST25) carrying the *bla*_OXA-23_ gene, confirming its circulation in the area (Rodríguez et al., 2016; Nodari et al., 2020). It should be noted that the characteristics of this clone are consistent with those previously described by our group in strains from the neighboring country, Bolivia (Cerezales González, 2018). Most isolates were assigned to IC2, a lineage known for its global distribution, although its presence in Latin-American countries is rarely reported (Castillo-Ramirez, 2023; Morgado et al., 2023), as in Brazil in 2008 and 2015-2016 (Rodríguez et al., 2016; Camargo et al., 2022). Within the isolates belonging to IC2, ST2 (Pasteur scheme) was the most prevalent sub-lineage, which is in accordance with the reported by other authors worldwide (Hamidian and Nigro, 2019). The most recent region-wide study also including isolates from Paraguay between July 2013 and June 2014, showed that the circulating clones belonged to IC5 (ST79 Pasteur) and IC7 (ST25 Pasteur), but no IC2 isolates were detected (Rodríguez et al., 2016). The presence of IC2 isolates carrying the *bla*_OXA-23_ gene in this study confirms the regional distribution of clone IC2, as described by other authors who identified this clone in South America (Levy-Blitchtein et al., 2018; Camargo et al., 2022; Martins-Gonçalves et al., 2024). During the COVID-19 pandemic, factors such as the massive influx of patients into ICUs and the increased use of antimicrobial agents intensified the selective pressure for multidrug-resistant pathogens. This led to outbreaks worldwide and also, in neighboring countries to Paraguay such as Brazil, where outbreaks caused by ST2 isolates, carrying *bla*_OXA-23_, were identified between 2020 and 2021 (Camargo et al., 2022).

The detailed analysis of the antibiotic resistance genes showed the presence of two *bla*_OXA-51_ variants, *bla*_OXA-66_ and *bla*_OXA-65_, with *bla*_OXA-66_ as the characteristic variant of the IC2 clone and in line with its emergence at a regional level (Camargo et al., 2022). As previously mentioned, all the isolates were carrying the carbapenemase gene *bla*_OXA-23_, a higher prevalence than the previously reported by Melgarejo et al (Melgarejo-Touchet et al., 2021). It is worth mentioning that IC2 isolates carried two copies of the gene, a frequent phenomenon among clinical *A. baumannii* isolates, according to the literature (Hua et al., 2016). These genes were located within transposons Tn*2006* and Tn*2008*, a commonly described structure that plays a key role in the overexpression and mobilization of the gene (Hamidian and Nigro, 2019; Sánchez-Urtaza et al., 2023). Additionally, two different variants of the *bla*_ADC_ gene were identified: *bla*_ADC-73_ and *bla*_ADC-5_. The isolates belonging to IC2 were carrying *bla*_ADC-73_ gene with IS*Aba1* upstream of it, a mechanism of overexpression that is commonly reported in clinical isolates (Nodari et al., 2020; Sánchez-Urtaza et al., 2023; Hamed et al., 2023). However, the *bla*_ADC-5_ gene that was identified in the isolate belonging to IC5 was accompanied by IS*3* upstream of it, which differs from the previously reported in other isolates from clone IC5 (Lopes et al., 2013).

Additional genes conferring resistance to other classes of antibiotics were also identified in isolates belonging to clone IC2: *armA*, *aph(6)-Id, aph(3’’)-Ib, msr(E), mph(E), sul1, catA1* and *tet(B)*; genes that have been reported recently in isolates that caused an outbreak in Brazil (Camargo et al., 2022). It is worth mentioning that in those countries where the IC2 isolates carrying the *armA* gene was detected and was predominant, they exhibited high resistance rates to aminoglycosides such as amikacin, gentamicin and tobramycin (Camargo et al., 2022; Martins-Gonçalves et al., 2024). In this study, the *armA, mph(E)* and *msr(E)* genes were identified in the chromosome within a Tn*6180*-derived fragment of the resistance island AbGRI3. This arrangement is globally spread and has been previously described in other CRAb isolates assigned to ST2 (Pasteur Scheme) and also ST195/1816 (Oxford Scheme), which is in accordance with the genetic contexts identified in our isolates (Wiradiputra et al., 2023; Wei et al., 2024). Although chromosomal integration may reduce the immediate risk of horizontal transfer compared to plasmid-borne elements, it can promote stable maintenance of these resistance genes within bacterial populations, and also, the presence of these genes in a Tn*6180*-derived structure suggests the potential for mobilization under selective pressure. This highlights the importance of monitoring chromosomal resistance islands as reservoirs of clinically significant resistance determinants. The *aph* genes (*aph(6)-Id, aph(3’’)-Ib*) together with *tet(B)* were found close to the carbapenemase gene *bla*_OXA-23_ in an AbGRI1-like resistance island in all the isolates belonging to IC2. This island has been repeatedly documented in clinical IC2 isolates across different countries and contributes significantly to their multidrug-resistant phenotype (Cerezales et al., 2020; Vijayakumar et al., 2022; Wiradiputra et al., 2023; Naderi et al., 2023). All the studied isolates belonging to IC2 were also harboring *sul1* and *catA1* together with *qacEΔ1* (antiseptic resistance) and *merC* (mercury resistance) genes in a complex structure of ~15 kb involving multiple mobile genetic elements such as IS*26*, IS*6100* and Tn*2*-like elements, that closely resembles mosaic resistance regions previously described in *Acinetobacter* genomic islands (Hamidian and Hall, 2018b). Moreover, IC2 isolates have been shown to carry AbGRI variants with class 1 integron backbones containing *sul1* and *qacEΔ1* together with IS26-mediated rearrangements (Harmer et al., 2023). These findings support the hypothesis that this region represents a chromosomally integrated resistance island related to AbaR or AbGRI structures that emerged through IS*26*‐mediated horizontal gene capture.

The resistome of the IC5 isolate also included a variety of genes conferring resistance to non-β-lactam antibiotics such as aminoglycosides (*armA*, *aph(6)-Id*, *aph(3’’)-Ib*, *aph(3’)-VIa*, *sat2* and *aadA1*), sulfonamides (*sul2*) and trimethoprim (*dfrA1*), which are commonly described in CRAb strains belonging to this clone (Nodari et al., 2020). In this isolate the *aph(6)-Id*, *aph(3’’)-Ib* and *sul2* genes were identified within a non-canonical variant of the transposon Tn*6250*, characterized by the insertion of an *ISAba125* element between the *traA* and *traD* genes. This transposon has been previously described by other authors (Brito et al., 2022), however, the presence of the IS*Aba125* was not previously reported. From a clinical perspective, such rearrangements may influence the mobility and transfer efficiency of resistance genes, potentially enhancing their spread across bacterial populations. The *aph(3’)-VIa* gene, is commonly associated with resistance to amikacin and is typically found within the Tn*AphA6* transposon, a composite element bracketed by IS*Aba125* insertion sequences (Khongfak et al., 2022; Brito et al., 2022). This transposon has been frequently identified in *A. baumannii* isolates, particularly those belonging to IC5, suggesting a strong association between this mobile element and the clonal lineage (Brito et al., 2022; Jouybari et al., 2021; Morgado et al., 2023). Moreover, Tn*AphA6* has been found both on conjugative plasmids and integrated into the chromosome, often in multidrug-resistant backgrounds (Khongfak et al., 2022; Brito et al., 2022). The mobility conferred by IS*26* likely facilitates its dissemination not only through clonal expansion but also via horizontal gene transfer. The presence of *aph(3’)-VIa* within the Tn*AphA6* transposon in our IC5 isolate further supports its role in the global spread of aminoglycoside resistance within this lineage. In addition, *dfrA1, sat2 and aadA1* genes were also identified together in isolate PR7 within a class 2 integron structure involving the *bla*_TEM-1_ gene. However, the additional insertion of a Tn*3* transposon harboring *bla*_TEM-1_ within the Tn*7* backbone, forming a Tn*7*::Tn*3* composite transposon, suggests active modular recombination events. This unusual arrangement may enhance the mobility and plasticity of Tn*7* by incorporating new resistance determinants. Similar nested transposition events have been reported for instance in ST79 *A. baumannii* isolates from Chile and Bolivia (Brito et al., 2022; Cerezales et al., 2020) but are relatively rare, and their presence highlights the role of Tn*7* as a key element for the acquisition of multiple resistance genes, particularly in multidrug-resistant *A. baumannii*.

Regarding the virulome of the isolates, most of the genes were involved in biofilm production, which is key for survival and invasion of host’s cells (Kumar et al., 2021). All the isolates were harboring genes coding for Csu fimbriae, BfmRS system, Bap protein, extracellular polysaccharide PNAG, OmpA, Ata transporter and AbaI/AbaR proteins, which according to the literature, are linked with the ability to form biofilm (Ghasemi et al., 2018). Another important virulence factor is the capsule polysaccharide K locus (KL) and outer core OC locus (OCL) (Müller et al., 2023). Capsular polysaccharide genes vary between isolates within the same species, and recent studies associate certain types of capsular polysaccharides or their alterations with increased virulence and biofilm production (Cahill et al., 2022). In this work, different capsule types were identified: OCL1/KL3, OCL1/KL81 and OCL10/KL9. Isolates belonging to IC2 had the OCL1 gene cluster, the most predominant among IC1 and IC2 according to the literature (Kenyon et al., 2014). Among the IC2 isolates, it is common to find a wide variety of different K loci, with KL3 gene cluster predominant according to other authors (Müller et al., 2023; Camargo et al., 2022; Cahill et al., 2022), which is in accordance with our results, where 4 out of 7 IC2 isolates were KL3. The OCL10 gene cluster is predominantly found in isolates belonging to IC5, as well as K locus KL9 (Müller et al., 2023), which is in accordance with our findings. Despite sharing identical virulence gene content and OCL/KL loci as other biofilm producing strains, isolate PR6 exhibited significantly reduced biofilm formation. A possible explanation is that this phenotype results from differences in the expression of biofilm-related genes, which are regulated by the BfmRS two-component system and the *csu* operon—both essential for pili-mediated surface attachment. Disruption of either *bfmR* or *bfmS* can lead to reduced *csu* expression, thereby preventing biofilm formation (Gao et al., 2019).

Plasmid analysis showed the presence of a RepAci1 plasmid of 8.73 Kb in all the isolates belonging to IC2. This plasmid has been described previously in isolates belonging to IC1, IC2 and IC3, encoding a toxin-antitoxin system (*brnT*/*brnA*), septicolysin and TonB-dependent receptor ZnuD2 (Aranzamendi et al., 2024; Hamidian and Hall, 2018a), but unlike some of those isolates, the Paraguayan isolates were not harboring any carbapenemase gene in their plasmids. In fact, some variants of r3-T1 plasmids carrying a carbapenemase gene appear to be limited to North America and Europe (Lam and Hamidian, 2024). Replicon typing experiments by PCR were positive for replicase Aci4, however, typing using Acinetobacter plasmid typing database published by Lam et al. (2023) did not show the presence of that replicase, instead, r3-T14 was identified, which is very similar to the r3-T11 (Aci4) and may explain the positive result in the PCR experiment (Lam et al., 2023).

The results obtained in this study allowed us to update and get an approximate picture of the current antibiotic resistance rates, resistome, virulome, plasmid profiles and also the circulating clones of *A. baumannii* in Paraguay. This type of study is crucial not only for gaining knowledge about the characteristics of the clones circulating in the country, but also to design and implement control measures to prevent outbreaks.

## Data Availability

The datasets presented in this study can be found in online repositories. The names of the repository/repositories and accession number(s) can be found in the article/**Supplementary Material**.
