# Supplementary material for: Genomic characterization of multidrug-resistant clinical Acinetobacter baumannii isolates from a hospital in Paraguay
Source: Front Cell Infect Microbiol. 2025 Jul 31;15:1620479. doi: 10.3389/fcimb.2025.1620479 (PMC12350285; doi:10.3389/fcimb.2025.1620479)
Supplement: Supplementary file 1 [file DataSheet1.docx]

Supplementary Material

**Genomic characterization of multidrug-resistant clinical *Acinetobacter baumannii* isolates from a hospital in Paraguay**

**Sandra Sánchez-Urtaza^1, 2, †^, Laura Alfonso-Alarcón^1,†^, Rocío Arazo del Pino^2,5^, Tessa Burgwinkel^2,5^, Alain Ocampo-Sosa^3^, Ruth Gonzalez^4^, Kyriaki Xanthopoulou^2,5^, Paul G. Higgins^2,5^*, Itziar Alkorta^6^, Lucia Gallego^1^***

*** Correspondence:**

Lucia Gallego*

lucia.gallego@ehu.eus

Paul G. Higgins*

paul.higgins@uni-koeln.de

# Supplementary Figures and Tables

## Supplementary Figures


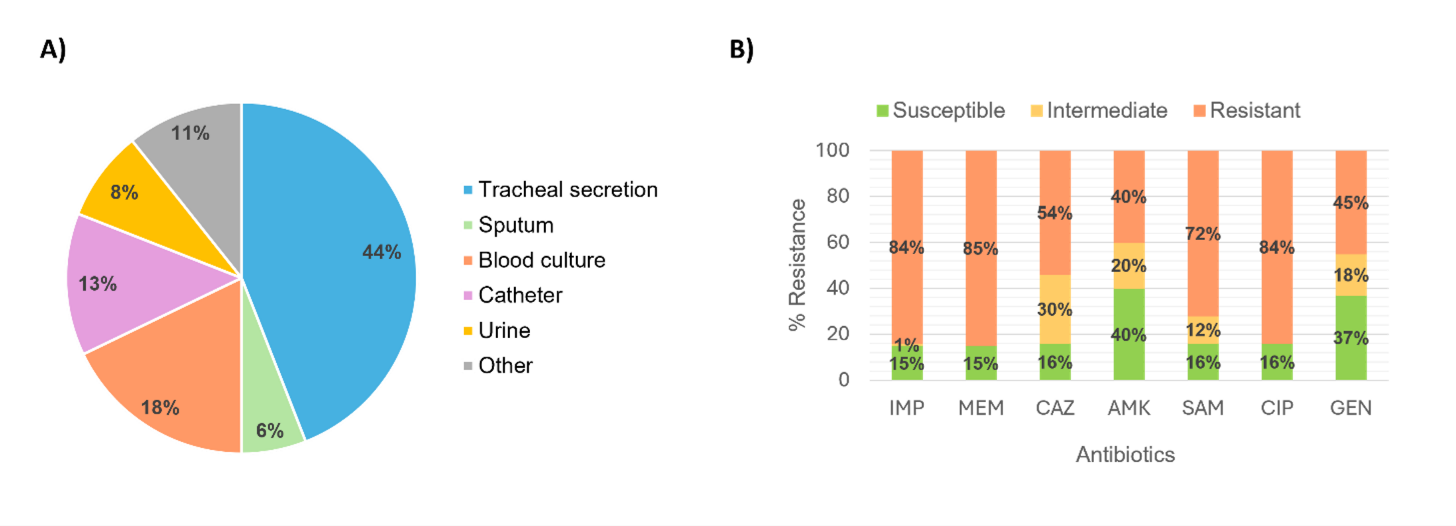


**Supplementary Figure 1.** A) Distribution of the type of clinical samples from where the *A. baumannii* isolates were recovered in 2022. B) Antibiotic susceptibility rates of the *A. baumannii* isolates collected in 2022. IMP: imipenem, MEM: meropenem, CAZ: ceftazidime, AMK: amikacin, SAM: ampicillin/sulbactam, CIP: ciprofloxacin, GEN: Gentamicin.


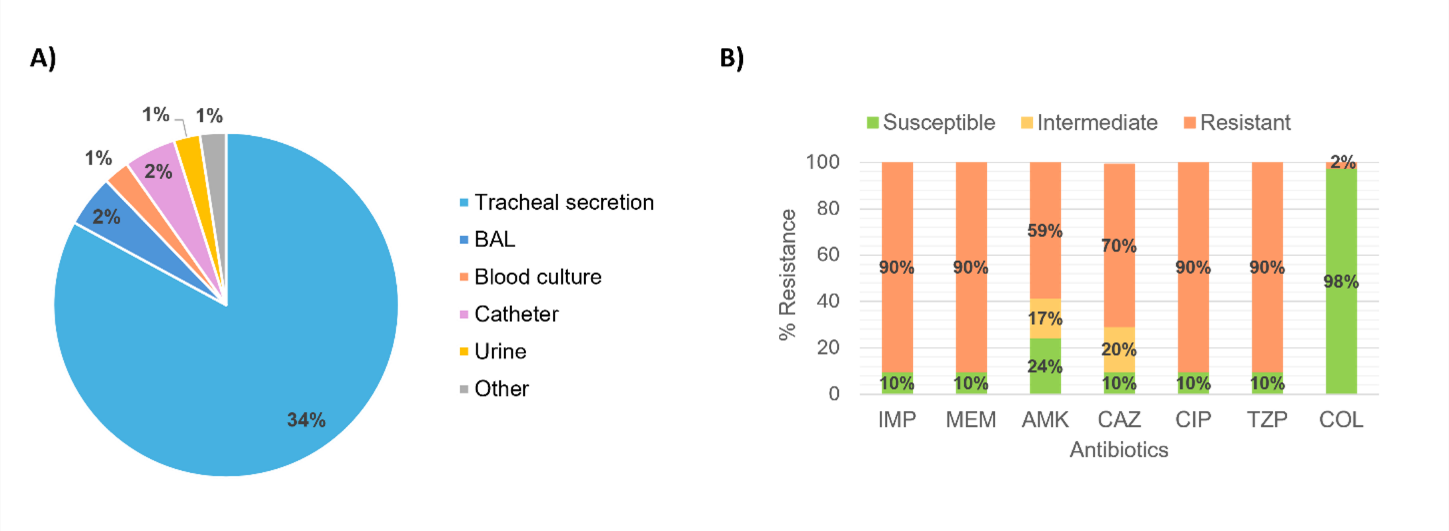


**Supplementary Figure 2.** A) Distribution of the type of clinical samples from where the *A. baumannii* isolates were recovered from January to February 2024. B) Antibiotic susceptibility rates of the *A. baumannii* isolates collected from January to February 2024. IMP: imipenem, MEM: meropenem, AMK: amikacin, CAZ: ceftazidime, CIP: ciprofloxacin, TZP: piperacillin/tazobactam, COL: colistin.


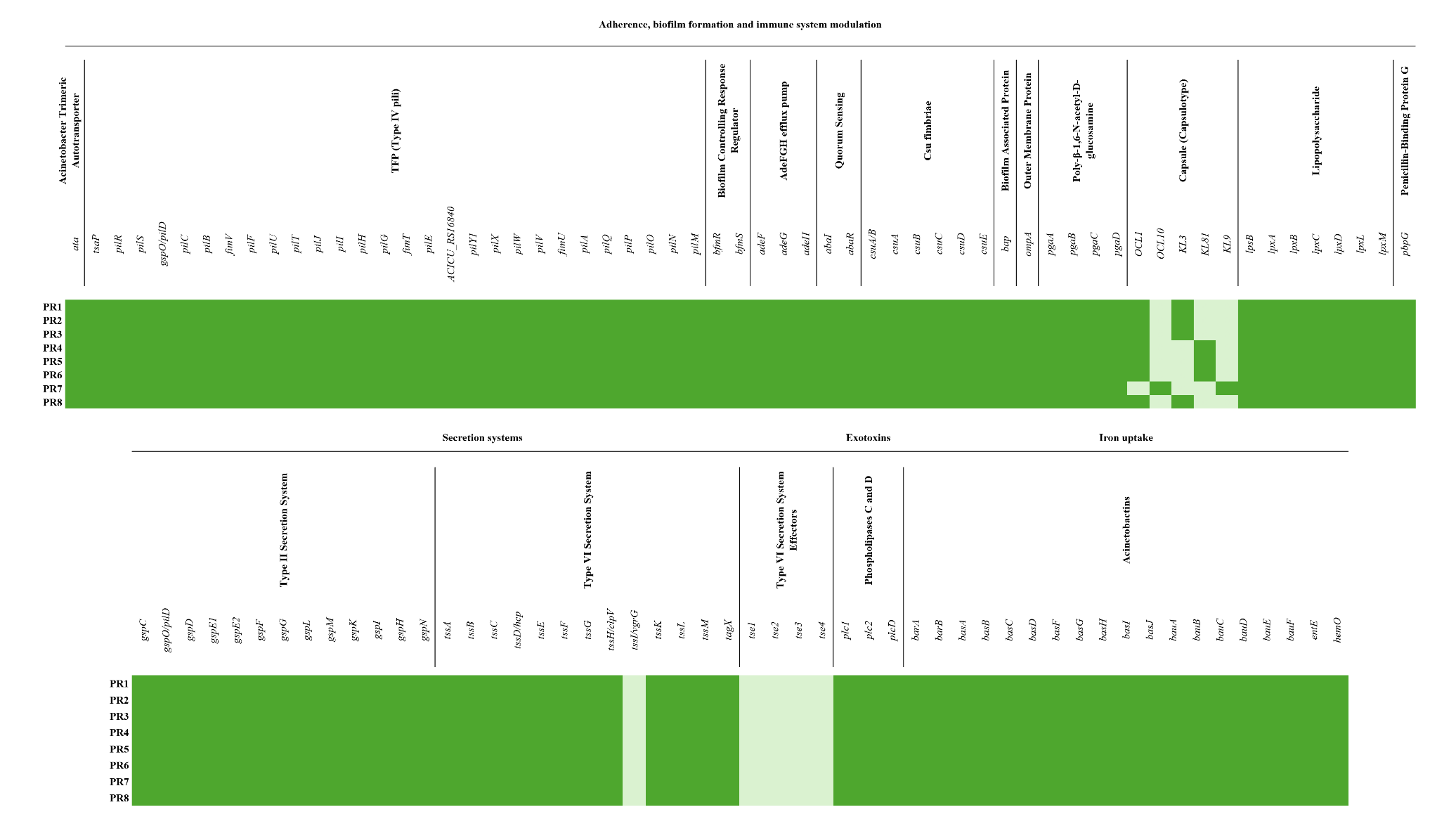


Supplementary Figure 3. Heatmap showing the presence of virulence-associated genes in *A. baumannii* isolates from the National Hospital of Itaugua (Paraguay). Dark green indicates the presence of the gene, while light green indicates its absence.


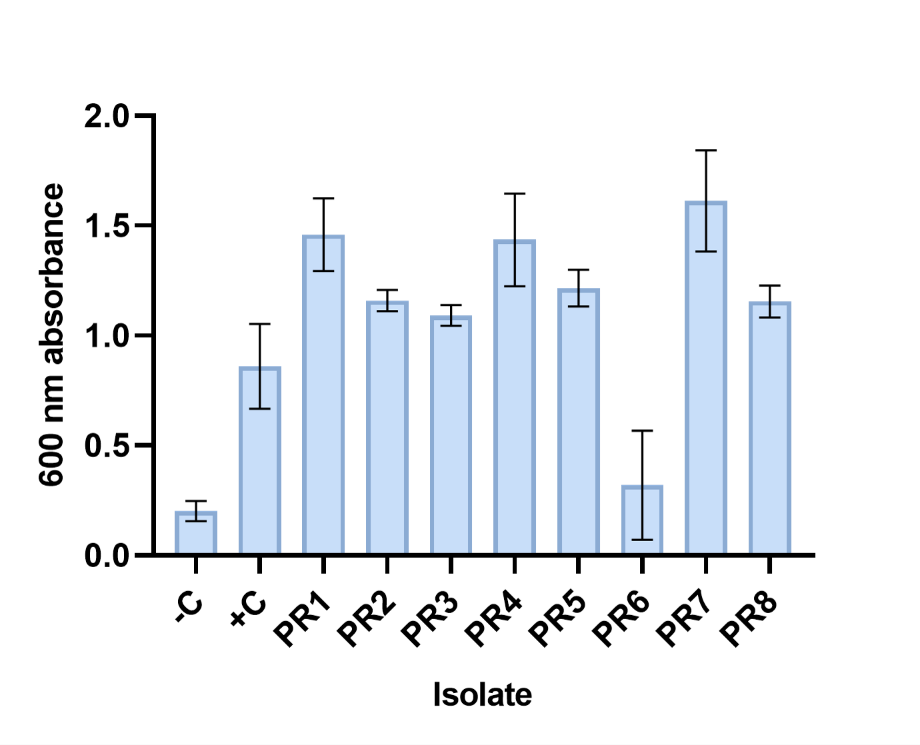


**Supplementary Figure 4.** Biofilm production measured as absorbance at 600 nm of the *A. baumannii* isolates. The isolates were compared using one-way ANOVA followed by Tukey's post hoc test. Statistically significant differences were considered at *p* < 0.05. Data are presented as mean ± standard deviation of three replicates. -C: negative control, +C: positive control.
